# Supplementary material for: Expansion and conversion of human pancreatic ductal cells into insulin-secreting endocrine cells
Source: eLife. 2013 Nov 19;2:e00940. doi: 10.7554/eLife.00940 (PMC3826580; doi:10.7554/eLife.00940)
Supplement: Supplementary file 1. — (A) Summary table of converted sphere transplantation. (B) Glucose-stimulated insulin C-peptide secretion in vivo. (C) PCR primers used for Ad-GFP-M6P construction. (D) Quantification data of cell immunostaining after FACS (Figure 1F). DOI: http://dx.doi.org/10.7554/eLife.00940.018 [file elife00940s001.doc]

**Supplementary file 1A. Summary of converted sphere transplantation**

| Site | Mouse ID | AV used | Sample Type | Ext. Media | Cell# (x10^6^) | Co-tranx Cells | Tranx dur. (days) | Serum human C-pept | Serum human Insulin | C-pept Cont. (pmol) | C-pept^+^ cells |
| --- | --- | --- | --- | --- | --- | --- | --- | --- | --- | --- | --- |
| K | 28 | 4V | Spheres |  | 0.9 | MEF | 14 | ND |  | 273 | NA |
| K | 29 | 4V | Spheres |  | 0.9 | MEF | 14 | ND |  | 126 | NA |
| K | 30 | 4V | Spheres |  | 0.9 | MEF | 28 | ND |  | 160^#^ | NA |
| K | 31 | 4V | Spheres |  | 0.9 | MEF | 28 | ND |  | 160^#^ | NA |
| K | 32 | 4V | Spheres |  | 0.9 | MEF | 14 | ND |  | 294 | NA |
| K | 33 | 4V | Spheres |  | 0.9 | MEF | 14 | ND |  | 422 | NA |
| K | 34 | 4V | Spheres |  | 0.9 | MEF | 27 | ND |  | 160^#^ | NA |
| K | 35 | 4V | Spheres |  | 0.9 | MEF | 27 | ND |  | 160^#^ | NA |
| K | 36 | 4V | Spheres |  | 0.9 | MEF | 16 | ND |  | 129^$^ | NA |
| K | 37 | 4V | Spheres |  | 0.9 | MEF | 16 | ND |  | 129^$^ | NA |
| K | 38 | 4V | Spheres |  | 0.9 | MEF | 16 | ND |  | 129^$^ | NA |
| K | 39 | 4V | Spheres |  | 0.9 | MEF | 16 | ND |  | 129^$^ | NA |
| K | 40 | 4V | Spheres |  | 0.9 | MEF | 16 | ND |  | 129^$^ | NA |
| K | 41 | 2V | Spheres | DF | 1.3 | MEF | 20 |  |  | 2704 | NA |
| K | 42 | 2V | Spheres | DF | 1.3 | MEF | 20 |  |  | 1012 | NA |
| K | 50 | 2V | Spheres | DF | 2.3 |  | 115 | ND | ND |  | YES |
| K | 51 | 2V | Spheres | DF | 3.4 |  | 103 | ND | YES |  | YES |
| K | 52 | 2V | Spheres | DF | 2.0 |  | 69 |  | ND | 1583 | YES |
| K | 53 | 2V | Spheres | T | 2.5 |  | 81 |  | ND |  | YES |
| K | 54 | 2V | Spheres | T | 5.8 |  | 49 |  | ND | ND | ND |
| K | 68 | 2V | Spheres | T | 6.0 |  | 151 |  | ND |  | YES |
| K | 100 | 2V | Spheres | T | 2.5 |  | 74 |  | YES |  | ND |
| K | 101 | 2V | Spheres | T | 4.0 |  | 74 |  | YES |  | ND |
| K | 113 | 2V | Spheres | DF | 0.8 |  | 1 |  |  |  | YES |
| K | 114 | 2V | Spheres | DF | 0.8 |  | 3 |  |  |  | YES |
| K | 115 | 2V | Spheres | DF | 0.8 |  | 7 |  |  |  | YES |
| K | 116 | 2V | Spheres | DF | 0.8 |  | 14 |  |  |  | ND |

**Supplementary file 1A. Continued**

| Site | Mouse ID | AV used | Sample Type | Ext. Media | Cell# (x10^6^) | Co-tranx Cells | Tranx dur. (days) | Serum human C-pept | Serum human Insulin | C-pept Cont. (pmol) | C-pept^+^ cells |
| --- | --- | --- | --- | --- | --- | --- | --- | --- | --- | --- | --- |
| K | 23 |  | Islets |  | 100(I) |  | 85 | YES |  |  | NA |
| K | 48 |  | Islets |  | 100(I) |  | 131 |  | YES |  | YES |
| K | 55 |  | Islets |  | 10(I) |  | 49 |  | YES |  | ND |
| K | 56 |  | Islets |  | 50(I) |  | 49 |  | YES |  | YES |
| EFP | 62 | 2V | Spheres | T | 8.0 |  | 182 |  | ND |  | ND |
| EFP | 111 | 2V | Spheres | DF | 3.8 |  | 29 |  | ND |  | ND |
| EFP | 112 | 2V | Spheres | DF | 3.8 |  | 29 |  | ND |  | ND |
| EFP | 108 | 2V | Spheres | DF | 6.7 |  | 41 |  | ND |  | ND |
| EFP | 109 | 2V | Spheres | DF | 6.7 |  | 41 |  | ND |  | ND |
| EFP | 110 | 2V | Spheres | DF | 6.7 |  | 39 |  | ND |  | ND |
| EFP | 58 |  | Islets |  | 10(I) |  | dead |  | YES |  | NA |
| EFP | 59 |  | Islets |  | 50(I) |  | 49 |  | YES |  | YES |
| Liver | 93 | 2V | Spheres | T | 1.5 |  | 6 |  |  |  | ND |
| Liver | 95 | 2V | Spheres | T | 1.5 |  | 29 |  | ND |  | ND |
| Liver | 97 | 2V | Spheres | T | 1.1 |  | 7 |  |  |  | YES |
| Liver | 99 | 2V | Spheres | T | 2.1 |  | 21 |  | ND |  | ND |

**Keys for Supplementary file 1A**.

| Column | Description |
| --- | --- |
| Site | Transplanted site. (K - Kidney, EFP - epididymal fat pad) |
| AV used | adenoviruses used for sphere conversion. (4V - MafA, Neurog3, Pdx1, Pax6; 2V - Neurog3, Ad-M6P) |
| Ext. Media | Culture media used for post-infection extended culture (see Materials and Methods for detail) |
| Cells | Number of cells transplanted (Number of IEQ for islets) |
| Co-tranx Cells | Type of cells co-transplanted with converted spheres (MEF - mouse embryonic fibroblast) |
| Tranx dur. (days) | Days from transplantation to graft harvest |
| Serum human C-pept | Presence of human C-peptide from graft in host blood serum (ND - not detected, YES - detected) |
| Serum human Insulin | Presence of human Insulin from graft in host blood serum (ND - not detected, YES - detected) |
| C-pept Cont. | Presence of total human C-peptide in harvested graft (ND - not detected; # - value obtained from pooled ID30,13,34,35; $ - value obtained from pooled ID36,37,38,39,40) |
| C-pept+ cells | Presence of C-peptide^+^/HuNu^+^ cells in harvested graft by IHC (NA - not available, ND - not detected, YES - detected) |

**Supplementary file 1B. Glucose-stimulated Insulin C-peptide secretion *in vivo***

| Animal ID | Site | Sample Type | Days post transpl. | C-peptide level (fasting) | C-peptide (pM, 30min. post gluc. inj.) | Insulin level (mU/L, fasting) | Insulin (mU/L, 30min. post gluc. Inj.) |
| --- | --- | --- | --- | --- | --- | --- | --- |
| 51 | Kidney | Sphere | 43 |  |  | 0.0841±0.0340 | 0.1666±0.0344 |
| 51 | Kidney | Sphere | 49 |  |  | 0.0814±0.0062 | 0.1236±0.0048 |
| 100 | Kidney | Sphere | 14 |  |  | ND | 0.0879±0.0042 |
| 101 | Kidney | Sphere | 14 |  |  | ND | 0.1209±0.0097 |
| 23 | Kidney | Islets (100) | 85 | 169.55±4.79 | 224.88±5.44 |  |  |
| 55 | Kidney | Islet (10) | 15 |  |  | 0.9350±0.0704 | 2.8669±0.0644 |
| 56 | Kidney | Islet (50) | 15 |  |  | 4.0315±0.0587 | 9.2035±0.1015 |
| 58 | EFP | Islet (10) | 15 |  |  | 0.3605±0.0101 | 0.5644±0.0179 |
| 59 | EFP | Islet (50) | 15 |  |  | 2.8040±0.0245 | 10.2234±0.0549 |

**Supplementary file 1C. PCR primers used for Ad-GFP-M6P construction**

| Primer Description | Primer Sequences |
| --- | --- |
| BglII-ATG-Myc-5’MAFA | ctcgaagatctatggagcagaaactcatctctgaagaggatctggccgcggagctggcgatgggcgccga |
| 3’MAFA-T2A-XhoI | ctgcatctcgagggggccggggttctcctccacgtcgccgcaggtcagcaggctgccgcggccctcgccgctgccgctgcgcttggcgcgcaggaagaagtcggccgtgcccttgg |
| XhoI-5’PAX6 | ggccccctcgagatgcagaacagtcacagcggagtg |
| 3’PAX6-HA-P2A-XmaI | cataggcccggggttttcttcaacatctcctgcttgctttaacagagagaagttcgtggcgccagatcctcgcttggcacgagcgtaatctggaacatcgtatgggtactgtaatcttggccagtattgaga |
| P2A-XmaI-5’PDX1 | gaaaaccccgggcctatgaacggcgaggagcagtactac |
| 3’PDX1-Flag-STOP-EcoRI | cactgggaattcctatcacttatcgtcgtcatccttgtaatctcgtggttcctgcggccgccgagg |

**Supplementary file 1D. Quantification data of cell immunostaining after FACS (Fi**gure 1F).

| **KRT19** | Total Cell # | Positive Cell # | Percentage (%) |
| --- | --- | --- | --- |
| CD133^+^ | 17083 | 16741 | 98.00 |
| CD133^neg^ | 9121 | 58 | 0.64 |
| Unsorted | 13275 | 3462 | 26.08 |
|  |  |  |  |
| **CPA** | Total Cell # | Positive Cell # | Percentage (%) |
| CD133^+^ | 16493 | 701 | 4.25 |
| CD133^neg^ | 7212 | 5296 | 73.43 |
| Unsorted | 12890 | 6056 | 46.98 |
|  |  |  |  |
| **C-peptide** | Total Cell # | Positive Cell # | Percentage (%) |
| CD133^+^ | 13764 | 0 | 0.00 |
| CD133^neg^ | 8353 | 16 | 0.19 |
| Unsorted | 11154 | 13 | 0.12 |
|  |  |  |  |
| **GCG** | Total Cell # | Positive Cell # | Percentage (%) |
| CD133^+^ | 13764 | 0 | 0.00 |
| CD133^neg^ | 8353 | 25 | 0.30 |
| Unsorted | 11154 | 25 | 0.22 |
|  |  |  |  |
| **SST** | Total Cell # | Positive Cell # | Percentage (%) |
| CD133^+^ | 16493 | 0 | 0.00 |
| CD133^neg^ | 7212 | 11 | 0.15 |
| Unsorted | 12890 | 7 | 0.05 |
|  |  |  |  |
| **PPY** | Total Cell # | Positive Cell # | Percentage (%) |
| CD133^+^ | 17779 | 0 | 0.00 |
| CD133^neg^ | 9008 | 20 | 0.22 |
| Unsorted | 11636 | 9 | 0.08 |
